# Pancreatic islet cell calcium ion imaging at single-cell resolution: functional identification of first-responder, highly connected (“hub”), and leader beta-cells

**DOI:** 10.3389/fendo.2026.1802510

**Published:** 2026-04-15

**Authors:** Luis Fernando Delgadillo-Silva, Giada Ostinelli, Audrey Provencher-Girard, Shadai Salazar, Rana Melhem, Guy A. Rutter

**Affiliations:** 1 Cardiometabolic Axis, University of Montreal Hospital Research Centre (CR-CHUM) and Department of Medicine, University of Montreal, Montreal, QC, Canada; 2 Section of Cell Biology and Functional Genomics, Division of Diabetes, Endocrinology and Metabolism, Faculty of Medicine, Department of Metabolism, Digestion and Reproduction, Faculty of Medicine, Imperial College London, Hammersmith Hospital, London, United Kingdom; 3 Lee Kong Chian School of Medicine, Nanyang Technological College, Singapore, Singapore; 4 Research Institute of the McGill University Health Centre, Westmount, QC, Canada

**Keywords:** beta-cells, Ca^2+^, connectivity, first-responders, hubs, islets, leaders

## Abstract

Increases in cytoplasmic free Ca^2+^ ions ([Ca^2+^]) are a critical signal in pancreatic islet beta-cells and are usually required for insulin secretion in response to glucose or other secretagogues. Changes in Ca^2+^, monitored using high-speed imaging across individual or multiple planes of the islet, can be used to explore the functional networks of beta-cells required for the precise regulation of insulin secretion. These networks are composed of functionally distinct beta-cell subpopulations: first-responders, highly connected hubs, and leader beta-cells, which initiate, connect, and dictate the pattern of spatially organized Ca^2+^ oscillations, respectively. Alterations in Ca^2+^ coordination among beta-cells contribute to defective insulin secretion, which underlies all forms of diabetes mellitus. Here, we provide a detailed protocol to perform Ca^2+^ imaging in isolated rodent islets, focusing on mouse islets expressing the genetic Ca^2+^ sensor, GCaMP6. We provide a step-by-step guide to evaluate general parameters of islet Ca^2+^dynamics, coordination, connectivity, and identification of specific functional subpopulations. This approach can be applied to investigate the role of Ca^2+^ dynamics and coordination in tissues where coordination is critical for normal function.

## Graphical Abstract

**Figure:**
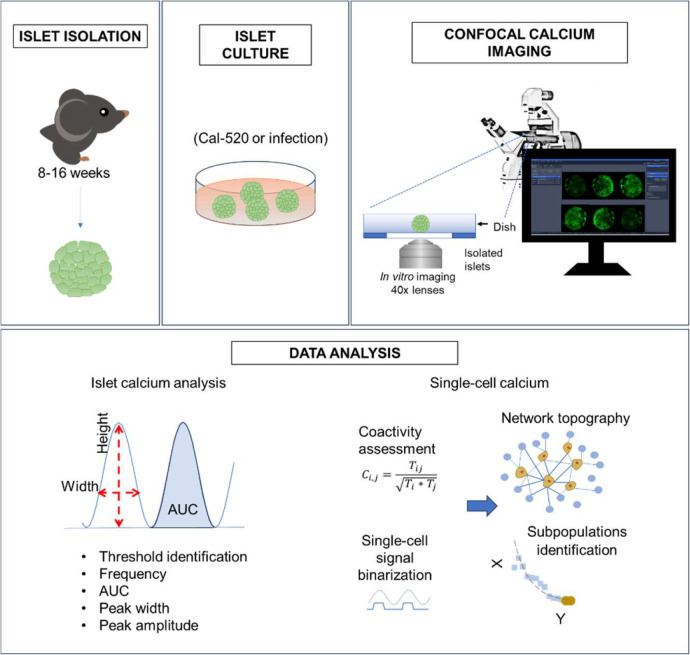

## Highlights

• Protocols are presented for islet isolation, culture, and multicellular imaging.
• Principles of islet beta-cell Ca^2+^ imaging using genetically encoded or intracellularly trappable Ca^2+^ sensors are described.
• We provide a pipeline for global islet-wide and single-cell analysis of Ca^2+^ dynamics.
• Approaches for the identification of functional subpopulations are described.

## 1 Introduction

Cellular communication is a fundamental property of tissues and is often required to coordinate the response to a stimulus across a large number of cells. In the heart, cardiomyocytes are electrically coupled via gap junctions. This enables these cells to quickly coordinate their function, in which a rise of cytoplasmic free Ca^2+^ triggers contraction (1). In the brain, neuronal circuits, such as the ones in the anterior lateral motor cortex, coordinate function through highly connected “hub” neurons, which are poorly influenced by the targeted area or the outcome reward, but serve as connection bridges for many cells (2). Similarly, pancreatic islets display a collective behavior to sense nutrients, proliferate, and secrete insulin (3, 4).

Pancreatic islets are micro-organs comprising ~1,000 cells that are dispersed throughout the pancreas (5). They are key regulators of blood glucose levels and are composed of glucagon-secreting alpha-cells, insulin-secreting beta-cells, somatostatin-secreting delta-cells, and pancreatic polypeptide-secreting gamma/pp-cells (6). Islets are found in humans, primates, rodents, and fish; however, the specific proportions of each cell type and islet architecture vary from one species to another. In rodents, beta-cells are found at the center and alpha-cells are localized at the islet periphery in a core–mantle organization. However, in zebrafish and humans, alpha-cells are dispersed through the islet (7). Irrespective of these differences, beta-cell coordination in response to glucose, in the form of spatially organized Ca^2+^ waves, appears to be a feature of all species so far examined (8–13).

All beta-cells are equipped with the molecular machinery to sense glucose and secrete insulin. Briefly, after a carbohydrate-rich meal, blood glucose levels rise. Glucose is taken up into the cells via glucose transporter GLUT2 in rodents, and via GLUT1, GLUT2, and GLUT3 in humans (14). Glucose is then phosphorylated by the critical glucose sensor glucokinase, and following glycolysis, it is metabolized by mitochondria. This increases the cytosolic ratio of ATP/ADP, which closes ATP-sensitive potassium channels (K_ATP_), triggering membrane depolarization and the activation of voltage-gated Ca^2+^ channels (VDCCs) (15). The resulting influx of Ca^2+^ increases the cytoplasmic Ca^2+^ concentration, which stimulates insulin secretion via exocytosis (16). On a multicellular scale, pancreatic beta-cells coordinate their cytoplasmic Ca^2+^ increases in the form of spatially organized Ca^2+^ waves. Localized changes in ATP/ADP close to K_ATP_ channels are also suggested to be important for their closure (17), though the importance of this mechanism is disputed (18).

Beta-cells were thought of historically as being a homogeneous population (19). This concept has evolved over the past 60 years, and it is now generally accepted that beta-cells are heterogeneous, for example, as revealed by single-cell RNA sequencing (RNA-seq) (20, 21). Beta-cells can also be classified as four antigenically distinct beta-cell subtypes by their differential expression of ST8SIA1 and CD9 on the cell surface (22). Furthermore, developmental beta-cells can stem from specific subpopulations of progenitors (21). Also, epigenetic control of DNA methylation (e.g., by DNMT3A) specifies Neuronatin (*Nnat*)-positive and -negative subpopulations during embryogenesis (22). The above studies have recently been reviewed by Rutter et al. (4). Nevertheless, existing -omic datasets present unavoidable limitations that interfere with direct comparison across studies (23). Moreover, -omic data usually represent a single snapshot and leave open the question of whether molecular heterogeneity reflects specific functional heterogeneity (24).

As mentioned above, following stimulation with high glucose, the islet demonstrates spatially organized intracellular Ca^2+^ waves that exhibit a complex behavior of rhythmic oscillations that can be broadly divided into the first and second phase (25, 26). We (27, 28) and others (9, 29–31) have shown that specific beta-cell subpopulations with distinct functional roles underlie islet Ca^2+^ dynamics: these subpopulations include “first-responders”, “leaders”, and highly connected “hubs”, comprising each 5%–10% of the overall beta-cell complement, and which together dictate islet Ca^2+^ dynamics. First-responders initiate the response to glucose during the first phase (31, 32). “Leaders” initiate Ca^2+^ waves while “hubs” connect the islet-wide network during the second phase or steady-state Ca^2+^ oscillations (15, 27, 33, 34).

While the reader is referred to Rutter et al. (4) for a more in-depth discussion of these points, the purpose of the present “Methods” article is to provide a step-by-step methodology to isolate islets, perform fast Ca^2+^ imaging, and quantify general islet-wide Ca^2+^ dynamics parameters, such as peak width, amplitude, frequency, and cumulative Ca^2+^ values [conveneiently referred to as an “area under the curve (AUC)”], followed by the identification of specific functional subpopulations based on their single-cell Ca^2+^ dynamics.

## 2 Islet isolation

We isolate islets according to either the protocol described in detail by Ravier et al. (35) or a modified protocol described in detail below (“A”), which is favored by one of us (LD). Both release 200–300 islets per mouse, or approximately 10% of the total available islets. Of note, other protocols for islet isolation have been published (36–38). However, in our hands, this method allows us to perform mouse islet isolation quickly and efficiently within ~40 min. In either case, mice of 6–20 weeks of age and from various strains (e.g., db/db and C57BL/6J) are usually used.

For the Ca^2+^ imaging studies described below, we usually use double transgenic mice: Ins1Cre: GCaMP6f^Flox/Flox^. The Ins1Cre line has Cre-recombinase knocked-in in the *Ins1* gene (39). The GCaMP6f^Flox/Flox^ cassette has been knocked-in at the Rosa26 genomic locus (The Jackson Laboratory, stock no. 028865). Double transgenic animals therefore express the genetically encoded Ca^2+^ indicator (GECI) GCaMP6f specifically in beta-cells (28, 40). The GCaMP family of sensors consists of a circularly permuted green fluorescent protein (cpGFP) linked to calmodulin (CAM) and the M13 peptide from the myosin light‐chain kinase (40, 41). At low Ca^2+^ levels, GCaMP shows low fluorescence, and upon Ca^2+^ binding into the CAM domain, the sensor undergoes a conformational change that dramatically increases its green fluorescence. Notably, late-generation green GECIs other than the GCaMP6 family exist. These include JGCaMP8 (42) and red GECI families including JRCaMP (43), JRGECO (44), and K-GECO (45) families, among others. Even far-red GECIs (FR-GECO) have been developed (46). These other sensors and chemical dyes can be employed together with this protocol; however, it might be necessary to adjust the activation threshold for each specific sensor.

### 2.1 Media and reagents

1. RPMI-1640 (Gibco™ 11875093) containing 11 mM glucose is referred to in this protocol as base-RPMI. To prepare complete-RPMI, proceed to supplement with 10% heat-inactivated fetal bovine serum (FBS), 100 IU/mL penicillin, and 100 μg/mL streptomycin. Store at 4 °C until use.
2. Prepare 4 mL per animal of Collagenase type IV from *Clostridium histolyticum* (800 CDU/mg) (Sigma C7667) by dissolving 1 mg/mL in Ca^2+^-free phosphate-buffered saline (PBS). Use fresh and keep it on ice until use.
3. Islets are separated from other pancreatic cells using density-gradient centrifugation employing Histopaque 1119 and 1077 (Sigma-Aldrich).
4. Instruments: stereoscope, tweezers, clamps, surgical scissors, 5-mL syringes, 30-G needles, 50- and 15-mL plastic tubes, centrifuge, and suspension Petri dishes.
5. Double transgenic mice Ins1Cre: GCaMP6f can be used to isolate the islets that express in beta-cell the genetically encoded Ca^2+^ indicator GCaMP6f. If not available, a chemical Ca^2+^ dye can be used as a surrogate (see 3 Islet culture, 3.2 procedure)

### 2.2 Protocol

**Figure 1:**
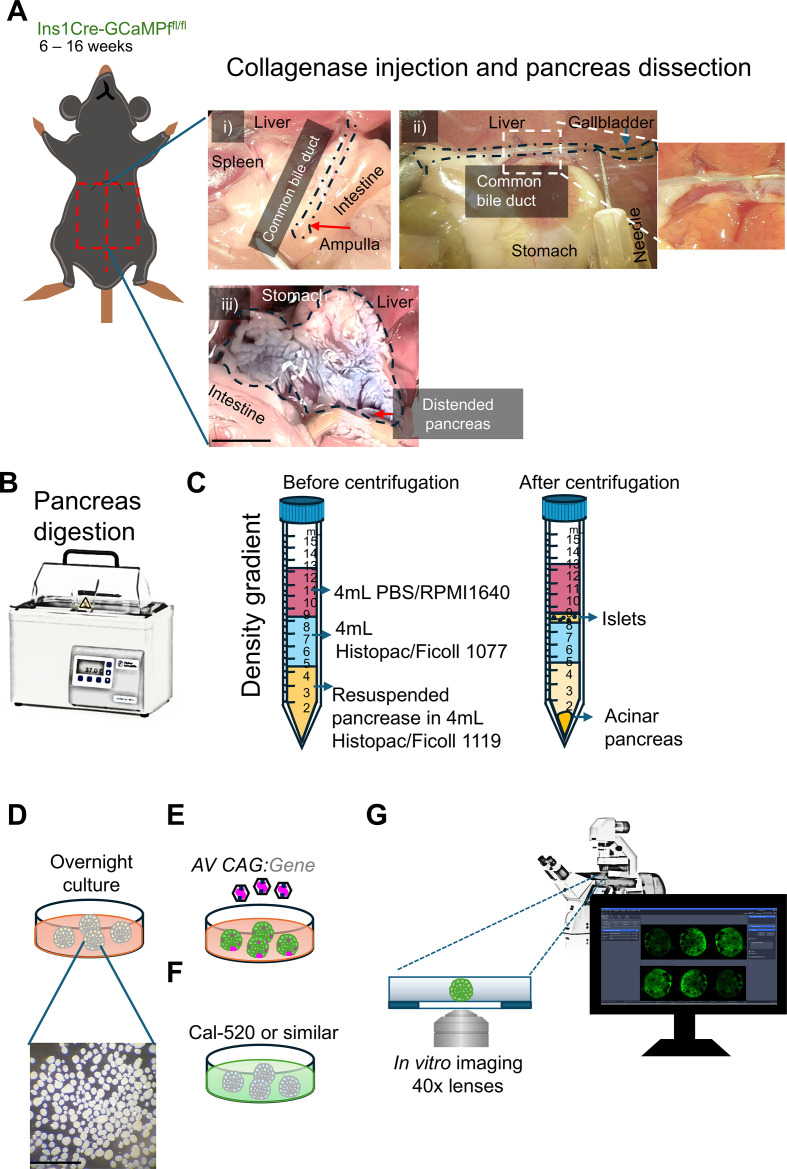
Islet isolation and culture. **(A)** Cartoon of a double transgenic Ins1Cre: GCaMP6f^Flox/Flox^ mouse. After deep anesthesia and cervical dislocation, the organs are exposed. (i) The common bile duct is exposed and, in the area of the ampulla, is clamped. (ii) Following the gallbladder, right after the bifurcation of the common bile duct, a small cut is done in one side of the duct and the needle is inserted. (iii) Carefully, the collagenase solution is delivered until the pancreas is distended. **(B)** After pancreas inflation and dissection, the pancreas is digested at 37 °C for 12 min. Then, the collagenase is neutralized. **(C)** The pancreas is centrifuged to separate the islets from the digested pancreas using a density gradient. **(D)** After recovering the islets from the histopaque, the islets are recovered into a new dish containing fresh media. **(E)** Islets that do not express the Ca^2+^ sensor (GCAMP6f, or similar) can be infected with adenovirus to express a GECI. **(F)** It is also possible to load the islet with chemical Ca^2+^ indicators such as Cal-520. **(G)** The isolated islet Ca^2+^ dynamics can be recorded using a confocal microscope.

1. Pre-cool the centrifuge to 4 °C.
2. After deep anesthesia, animals should be killed by cervical dislocation, or according to the locally approved ethical protocols.
3. Spray and soak the fur with 70% ethanol. Using tweezers, pull up the lower skin of the abdomen, and with the surgical scissors, cut the skin along the abdomen until reaching the sternum. Expose the internal organs and locate the pancreas around the guts and stomach. Find the common bile duct connecting the liver, pancreas, and guts (**Figure 1Ai**).
4. Under a stereoscope, and using a 2× magnification, move the liver upward, and move other organs aside using tweezers to expose the pancreas located under the stomach and around the guts. Identify the gallbladder around the liver. Move the liver to expose the extrapancreatic duct. Following the extrapancreatic duct towards the small intestine, move out gently the rest of the organs gently to properly expose the duct leading from the gallbladder into the liver up to the intestine (**Figure 1Ai**).
5. Fill a 5-mL syringe with 4 mL of collagenase solution. Attach a 30-G needle to the syringe. Using tweezers, bend the needle in the middle to ~90°.
6. Follow the gallbladder and locate the bifurcation where the gallbladder and the liver ducts fuse (**Figure 1Aii**). With small surgical scissors, make a small superficial cut on only one side of the duct, to enable easier insertion of the needle. Do not cut through the whole duct, as the needle insertion becomes cumbersome.
7. Slowly insert a 30-G needle, inside the small cut previously made. Slide the needle inside the bile duct toward the pancreas around 2–5 mm (**Figure 1Aii**). Slowly deliver ~3–4 mL of collagenase solution (1 mg/mL) into the pancreatic duct until the pancreas is fully distended (**Figure 1Aiii**). Note: The collagenase solution should be kept on ice during the procedure.
8. After pancreas distention with collagenase (**Figure 1Aiii**), proceed to dissect the pancreas along the intestine up to the stomach using small surgical scissors. Finally, remove the spleen from the pancreas and recover the pancreas in a 50-mL falcon tube. If several mice are sacrificed, separate each pancreas in tubes and keep on ice for a maximum of 1 h.
9. To digest the pancreas, transfer the tubes containing the pancreas into a 37 °C water bath for 12 min (**Figure 1B**). After the first 10 min, shake the tubes vigorously 10 times and put them back into the water bath and digest for a further 2 min. Note: Extending the digestion time beyond 12 min should be avoided as this will also digest and break the islets.
10. Stop the collagenase digestion by filling the tubes with up to 25 mL of base-RPM1-1640. Shake vigorously 10 times, centrifuge at 462 g for 3 min, and discard the supernatant to wash out the collagenase. Note: All solutions in contact with the pancreas must be neutralized with chlorine bleach (usually 10%) or a suitable solution according to the institutional regulations.
11. Resuspend the pellet in 4 mL of histopaque solution (1.119 g/L) and transfer the solution containing the digested pancreas and islets into a 15-mL falcon tube. Carefully add dropwise the next layers of 4 mL of histopaque (1.077 g/L) and finally add 4 mL of base-RPMI-1640, for a total of 12 mL and three layers (**Figure 1C**).
12. Centrifuge at 339 g for 20 min at 4 °C.
13. Recover the first two layers (base-RPMI-1640 and histopaque 1.077 g/L) in a 15-mL suspension Petri dish containing 10 mL of base-RPMI-1640.
14. Under a stereoscope using 4× magnification, the islets are opaque spheres ranging from 50 to 500 μm (**Figure 1D**). With a pipette and filter tips, proceed to handpick the islets and transfer the islets into a new sterile 15-mL suspension Petri dish with 12 mL of complete RPMI-1640. The islets are cultured for a maximum of 2 days at 37 °C in a humidified incubator with 5% CO_2_. Media (complete RPMI-1640) can be changed every 2 days of culture. Note: It is recommended to use the islets for experiments immediately after an overnight cell culture. Insulin secretion and Ca^2+^ dynamics are diminished over time.

### 2.3 Comments

During islet isolation, full pancreas distention via the delivery of the collagenase solution is the single most critical step for efficient islet isolation.

## 3 Islet culture

### 3.1 Media and reagents

1. Twelve-well suspension plates, complete RPMI-1640 (see 2 Islet isolation).
2. After overnight recovery, islets can be transduced using adeno-associated viruses (AAVs) or adenovirus (AV), to express a genetic Ca^2+^ sensor such as GCaMP (**Figure 1E**). Usually, islets are infected using a multiplicity of infection (MOI) of 30–100. However, it is typical to test several MOIs to maximize infection and reduce mortality (47). Note: viral manipulation usually requires N2-level containment; proceed according to institutional guidelines.
3. Four to 24 h post-infection, transfer the infected islets to a new 12-well suspension plate containing 1 mL of fresh complete RPMI-1640 medium (47). If the islets are from transgenic mouse lines such as Ins1Cre: GCaMP6f, which express a Ca^2+^ sensor specifically in beta-cells, they can be already imaged. Note: Initial expression is usually visible after 24 h post-infection; however, it is recommended to verify the expression levels at different time points (24, 36, or 48 h).
4. Islets from backgrounds that do not express a genetically encoded Ca^2+^ indicator (GECI) can also be loaded with a chemical Ca^2+^ sensor such as Cal-520 (**Figure 1F**; AAT-Bioquest Cat 21130).

### 3.2 Procedure

1. For viral transduction, transfer 10–20 islets into a 12-well suspension plate in 1 mL of complete RPMI-1640. Then, add the volume of virus with a MOI of 30–100 (determined by the user), and after 4–24 h of infection, wash three times by picking up the islet with ~10 μL of media and using filter tips and transferring them to a new 12-well suspension plate with 2 mL of fresh complete RPMI-1640. Neutralize all solutions and tips used during viral infection and wash islets with 10% chlorine bleach or a suitable solution according to institutional regulations.
2. A chemical Ca^2+^ sensor such as Cal-520 can be used to monitor Ca^2+^ dynamics. Briefly, prepare a 2 mM stock solution in DMSO. Dilute to a final concentration of 2–5 μM in 1 mL of media and add to a final concentration 0.04% Pluronic^®^ F-127 (optional) (AAT-Bioquest Cat 20053). Incubate 10–20 islets at 37 °C for 1 h. Transfer the islets into a new 12-well suspension using a pipette and 20-μL filter tips, with 2 mL of fresh complete RPMI-1640.
3. Proceed with Ca^2+^ imaging (**Figure 1G**).

### 3.3 Comments

We have noted that islet Ca^2+^ dynamics changed over the time the islets are cultured (48). If the islets need to undergo treatments such as glucolipotoxicity or AV infections, which extend the time in cell culture, appropriate time-matched controls should be employed.

## 4 Ca^2+^ imaging

### 4.1 Media and reagents

1. Inverted confocal microscope with 40× oil objective, equipped with an incubation system for temperature control (37 °C) and laser lines including 488 nm for “green” Ca^2+^ sensors such as GCaMP and Cal-520. Note: There are other “red” GECI and chemical Ca^2+^ indicators that might require other laser lines such as 561 and 640 nm (46).
2. Poly-D-lysine (0.1 mg/mL) (Gibco, A3890401).
3. Laboratory pipettes and tips (1,000, 200, and 20 μL).
4. 35-mm glass-bottomed Petri dishes (MatTek, P35G-1.5-10-C, with 1.5 coverglass, 0.16–0.19 mm)
5. Twelve-well suspension plates, complete RPMI-1640 (see 2 Islet isolation).
6. Prepare HEPES-bicarbonate (modified-Krebs, referred to as Krebs or Krebs buffer solution) buffer solution, CO_2_, pH 7.4 (**Table 1**).
7. Glucose solution (1 M) dissolved in Krebs.
8. KCl solution (1 M) dissolved in Milli-Q H_2_O.

**Table 1:** Reagents and concentration required for preparing HEPES-bicarbonate (modified Krebs) buffer solution.

Mixed salt solution				
reagents	1× Conc	4× Conc	MW	1 L (5×) (g)
NaCl	130 mM	650 mM	58.44	37.98
KCl	3.6 mM	18 mM	74.55	1.342
NaH_2_PO_4_	0.5 mM	2.5 mM	137.9	0.345
MgSO_4_	0.5 mM	2.5 mM	246.48	0.616
CaCl_2_	1.5 mM	7.5 mM	147.02	1.102

Bicarbonate solution				
reagents	1× Conc	4× Conc	MW	1 L (4×) (g)
HEPES	10 mM	40 mM	238.31	9.53
NaHCO_3_	24 mM	96 mM	84.01	8.064
NaCl	10 mM	40 mM	58.44	2.34

The first column indicates the component, followed by the final concentration at 1× and stock solution. The third column indicates the molecular weight of each component. The last column indicates the amount required expressed in grams to make a stock solution.

### 4.2 Procedure

**Figure 2:**
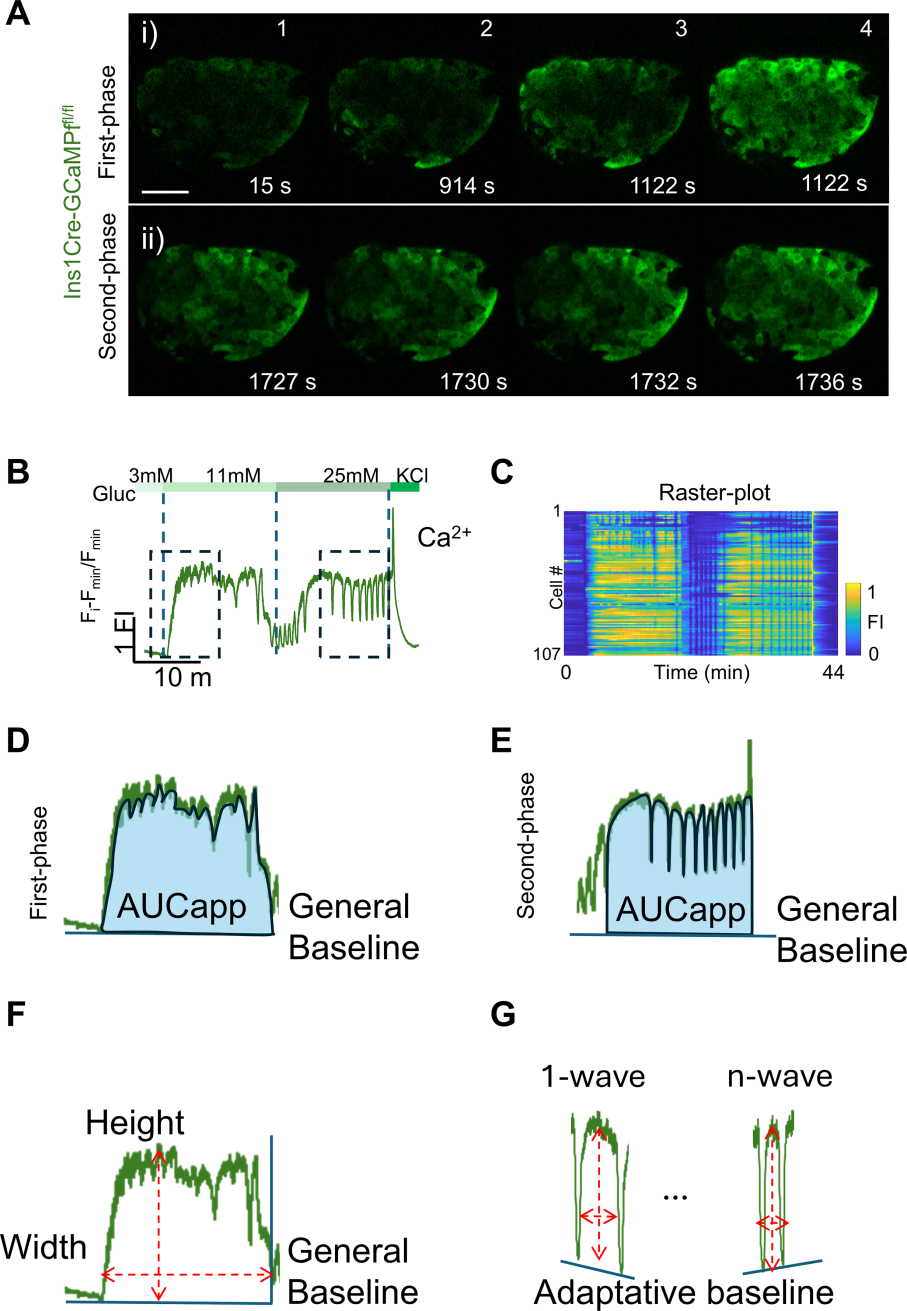
Islet Ca^2+^ imaging. **(A)** Snapshots from confocal images of an isolated islet (**Movie S1**). (i) During the first phase, or exposure to 11 mM glucose, and (ii) during exposure to 25 mM glucose. **(B)** Calcium fluorescent trace from the islet shown in **(A)**. **(C)** The rasterplot shows the activity of individual cells from the islet shown in **(A)**. **(D)** The AUC is represented by the blue-colored area under the islet fluorescent trace, specifically during the first phase and using the general (3 mM) recorded baseline. **(E)** The AUC depicted, specifically during the time after increase of glucose to 25 mM or second phase and using the general (3 mM) recorded baseline. **(F)** The first-phase peak height and width are denoted by the red dotted lines. **(G)** Individual peaks are shown and how to identify the peak height and width of each individual Ca^2+^ wave during the second phase by deploying an adaptative baseline.

1. Prepare HEPES-bicarbonate (Krebs buffer solution). Briefly add 100 mL of 5× mixed salts buffer, 125 mL of 4× bicarbonate solution, and 275 mL of Milli-Q water (see Supplementary Materials). Bubble CO_2_ 15–20 min, if not keep in a cell culture incubator for 1–2 h. Using a potentiometric pH meter, measure the initial pH, and dropwise adjust the pH to 7.4. Stir constantly the solution using a magnetic stirrer during pH adjustment.
2. Keep at 37 °C until use. Add glucose to the Krebs solution to reach a final concentration of 3 mM.
3. Move 5–10 islets to a 12-well suspension plate with 1 mL of Krebs containing 3 mM glucose. The islets should be in 3 mM or low glucose for at least 30–45 min. To achieve high-resolution imaging, select islets that do not present any signs of physical disruption or any residual acinar tissue adherence.
4. To help minimize islet movements during imaging, we suggest the use of poly-D-lysine, which is an extracellular matrix that facilitates islet adherence to the glass and prevents them from moving during the imaging. First, coat the glass-bottomed Petri dish (MatTek P35G-1.5.10C) with 300 μL of poly-D-lysine (0.1 mg/mL) for 10 min, then remove the poly-D-lysine solution and wash once with PBS and remove all liquid.
5. Add immersion oil or imaging immersion media required on the objective.
6. Position the coated glass-bottomed Petri dish on the microscope stage equipped with the incubation system. Add 2 mL of Krebs 3 mM glucose with a pipette. With the 20-μL pipette, take the islets from the 12-well suspension and carefully deliver three to five islets into the center of the glass-bottomed Petri dish. Since the glass surface is coated with poly-D-lysine, the islets will quicky adhere to it. We suggested to start with 5–10 islets, some of which might be lost during transfers. Similarly, if an islet does not reach the center of the Petri dish and end up into the rim, it might not be accessible for imaging. If several islets are successfully loaded, randomly select one for imaging.
7. Set up the microscopy acquisition parameters to obtain <2 images/s (<2 Hz) on a single plane of 512 × 512 pixels—153 × 153 μm (scaling per pixel < 0.3 μm × 0.3 μm). Set up the pinhole at 1 Airy unit. Use the 488-nm laser line for GCaMP6f excitation and a detection window of 498–540 nm. Use time series for a total time of 42 min, or 5,040 frames, with an acquisition speed of <500 ms per frame. Adjust the detector gain and power laser to cover ~5%–10% of the color histogram.
8. Start the imaging video, after 360 frames or 3 min; using the 20-μL pipette, deliver 16 μL of 1 M glucose solution to a final concentration of submaximal-stimulatory glucose of 11 mM. At 2,160 frames or 15–20 min of imaging (can be extended to provide extra time for connectivity analysis at 11 mM), increase glucose to a maximal 25 mM concentration by adding 28 μL of 1 M glucose solution, using the 200-μL pipette. Finally, force membrane depolarization by adding 80 μL of 1 M KCl solution into the dish to a final 40 mM KCl (**Figures 2A–C**). Of note, the 11 mM glucose time can be extended to 30 min if connectivity should be assessed during this glucose concentration.
9. Save the image file.

### 4.3 Comments

It is necessary to keep the laser power as low as possible (<1.5%) to minimize phototoxicity. Of note, GCaMP6 sensors have a dynamic range, defined as the maximum fluorescent intensity divided by the minimum fluorescence (*F*_max_/*F*_min_) of ∼63-fold increase upon Ca^2+^ binding (40). Therefore, it is also important to not set too high the sensitivity of the detectors, as these can be saturated, which will interfere with further quantifications. During imaging, it is also important to avoid touching the plate with the tips while delivering the solutions as this can interfere with imaging. This protocol suggests a “low-submaximal-maximal-depolarization” procedure. However, a return to low before the addition to KCl can be included. This might be useful for exploring formation and dispersion of islet functional networks (49). This can be achieved by coupling this method with a perifusion system to provide a dynamic range of glucose, or addition of drugs, which is not limited to the conditions here suggested. The typical first peak lasts for 3–10 min, before steady-state Ca^2+^ oscillations initiate (**Figure 2B**) (50).

## 5 Islet global analysis

### 5.1 Islet and single-cell fluorescence signal extraction

1. During Ca^2+^ imaging, the addition of glucose achieved by direct bath application using a pipette might cause small, but significant, changes in the focal plane causing *XY*- or *Z*-drift. Islet displacement into the *XY*-dimensions can be easily corrected using ImageJ (https://imagej.net/software/fiji/). Load the image file to analyze into ImageJ. Then, correct the *XY*-movements by using the plugin “Descriptor-based series registration (2d/3d + t)” (https://zenodo.org/records/14042795 (2d/3d)], applying the model “Rigid (2d)” with “3-dimensional quadratic fit” (51). For *Z*-movements, it is either manually or automatically corrected by quickly refocusing the *Z*-plane during the acquisition. Under these conditions, the impact of small *Z*-movements can be minimized by smoothing the Ca^2+^ signals with a moving average filter after fluorescent trace extraction (see below). Of note, if the cell moves out of the *Z*-plane for longer than a couple of frames and is not successfully refocused, this cell should not be analyzed.
2. To extract the fluorescent traces, in ImageJ and using the Polygon tool, circulate the islet and add the polygon to the region of interest (ROI) manager. Save the ROI. Go to “Analyze”, “Set measurements”, and select only “Integrate density” (i.e., total fluorescence of selected ROI). From the ROI manager plug-in, click on “more” and click on “Multi Measure”. This will all measure the fluorescence from all frames. Save it as a csv file.

### 5.2 Smoothing, baseline subtraction, and noise reduction

1. After extraction of the fluorescent values, the noise can be reduced by “smoothing” the fluorescent trace. Using MATLAB, smooth the islet fluorescent trace using a moving-average filter, with a size of 3–15 frames (simply use the function “smooth” from MATLAB using as arguments the fluorescent trace, and filter size: 15). This helps reduce the noise and maximize the signal. However, over-smoothing can mask small peaks. After smoothing, subtract the general baseline. The general baseline corresponds to the minimum value recorded at 3 mM glucose. Subtract the baseline from all the fluorescent values, and then divide it by itself, to express the fluorescence as “Fold change” using the following formula:


$$\mathrm{Fold change}=\frac{F_{i}-F_{\text{min}}}{F_{\text{min}}}$$

where *F_i_* is the fluorescence at a given time. *F*_min_ is the minimum intensity during the Ca^2+^ recordings. The expression of the fluorescence signal as a fold change helps to distinguish noise from signal.

### 5.3 Area under the curve

Two approaches can be used to estimate changes in intracellular free Ca^2+^ concentration over time. It must be noted, however, that both represent approximations given that GCaMP6 fluorescence values are uncalibrated in terms of absolute intracellular free Ca^2+^ concentrations.

1. An “apparent area under the curve” (AUC_app_) can be estimated by summing fluorescence data over time (LD). ImageJ is used to extract the fluorescent traces and MATLAB for baseline subtraction and to provide smoothing (see above). It is then possible simply to sum the fold change fluorescence values over a defined period required to calculate an AUC_app_. Of note, the minimum value over the fluorescent trace should be 0 (**Figures 1A–C**). The AUC_app_ calculations can be separated into specific sections, and therefore, the AUCapp of the first phase and second phase can be individually quantified (**Figure 2D**).


$$\mathrm{AUCapp}=\sum_{t=i}^{j}(F_{i})\;$$

where *F_i_* is the integrated fluorescence, expressed as a fold change (see above) at a given time and up to time *j*.

2. A more formal, and mathematically rigorous, calculation of AUC can be achieved by applying the “trapezoid rule” (https://www.graphpad.com/guides/prism/latest/statistics/stat_area_under_the_curve.htm) (GO). Here, the AUC calculations can be separated into specific sections, and therefore, the AUC of the first phase and second phase can be individually quantified (**Figure 2D**).


$$\mathrm{AUC}=\sum_{t=i}^{j}(F_{i}-F_{i+1})*({\mathrm{Time}}_{i}-\;{\mathrm{Time}}_{i+1})/2\;$$

where *F_i_* is the integrated fluorescence, expressed as a fold change (see above) at a given time and up to time *j*.

To quantify the AUC of the second phase, simply apply the same steps and quantify after the addition of 25 mM glucose (**Figure 2E**).

### 5.4 First-phase peak amplitude and width

1. The first-phase peak amplitude is the maximum value obtained during the first and usually highest fluorescent peak during the first 3–10 min after glucose stimulation. Simply, find the maximum value of the first-peak fluorescent trace. This is the first-peak amplitude and indicates the “strength” of first-peak response (**Figure 2F**).

$$\text{Amplitude}=\text{max}(F_{i}),\;t=0\;\text{min},\;t=15\;\text{min}$$

2. To calculate the first-phase width, simply find the time point when the first-peak fluorescent trace increased over 20% fold change baseline, or T_20_ after the increase of glucose from 3 to 11 mM. This value corresponds to the activation time of the first-phase T_20_ or FP-T_20_. The first-phase end is detected as a significant decrease of fluorescence. Then, the islet transitions to a complex behavior of steady-state Ca^2+^ waves. The time from activation to the next oscillation or significant activity decrease is considered as the peak width (**Figure 2F**).

### 5.5 Second-phase frequency, peak amplitude, and peak width

**Figure 3:**
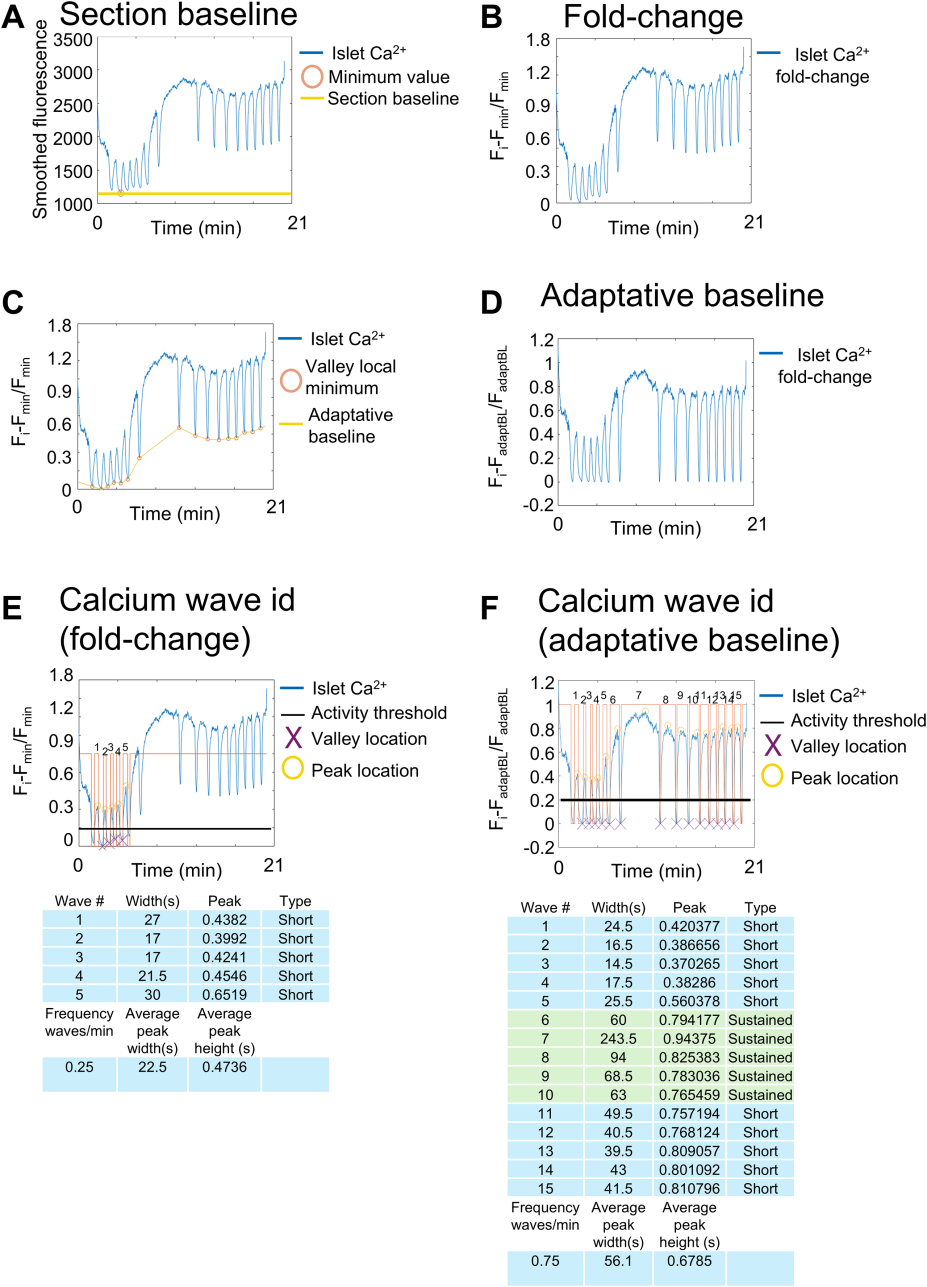
Baseline detection on fluorescent traces. **(A)** Calcium fluorescent trace from the islet shown in **Figure 2A** after smoothing. The orange circle depicts the section minima that is used to subtract the specific section baseline and expressed as a fold change. **(B)** Calcium trace depicted as a section fold change. **(C)** Section islet Ca^2+^ fold change trace, showing the valley identification (orange circles) and adaptative baseline calculation (yellow line). **(D)** Re-expressed Ca^2+^ trace depicted as a section fold change after adaptative baseline subtraction. **(E)** Fold-change Ca^2+^ trace, showing the identification of individual Ca^2+^ waves by imposing an activity threshold of 20% over the baseline (black line) and sustained activity for at least 2 s (peak width, the orange line). The maximum value during individual Ca^2+^ waves is referred to as Ca^2+^ wave peak (yellow circle). **(F)** Fold-change Ca^2+^ trace after adaptative baseline subtraction and showing individual Ca^2+^ waves, similarly to **(E)** by imposing an activity threshold of 20% over the baseline (black line) and sustained activity for at least 2 s (peak width, the orange line). The maximum value during individual Ca^2+^ waves is referred to as Ca^2+^ wave peak (yellow circle). Calcium waves can be broadly divided into short (<1 min) and sustained (>1 m).


1. After an initial increase to 11 mM glucose, it is common for the Ca^2+^ baseline to not return to the original baseline at 3 mM. This can also occur after the increase to 25 mM glucose. Therefore, to account for the changes in the Ca^2+^ baseline and properly identify Ca^2+^ oscillations, an adaptative baseline can be calculated over a specific section (compare the baseline in **Figures 2F, G**). This helps to detrend and correct changes in the baseline over time and facilitate the identification of individual Ca^2+^ waves. Other methods include utilizing Huang-Hilbert filtering (33). To perform this step and analyze the second phase either during the 11 mM glucose or after increasing the glucose to 25 mM, use the smoothed fluorescent trace (see above 5.2, **Figure 3A**), and as an independent section to analyze, express it as a fold change (see above 5.2). The *F*_min_ for this section marks the baseline for the selected section (**Figure 3B**).
2. To generate the islet adaptative baseline, further apply the “findpeaks” function using MATLAB, using as arguments the fold-change islet fluorescent trace, for the identification of both peaks and valleys. Then, remove all the peaks too close to the valleys, i.e., <25 frames or 12.5 s. This is because a Ca^2+^ wave starts to deactivate, and the signal usually goes back in ~12.5 s. Proceed to remove non-significant valleys, or those that do not present at least a 30% decreased decrease invalue, in comparison to the immediately preceding peak; i.e., keep only valleys satisfying: (Peak−Valley)/Peak ≥ 0.30; of note, MATLAB findpeaks provide the location and value for both, peaks and valleys. Finally, connect the n-valleys using the equation of straight line equation:

y = mx + b

where the first point is the valley*_i_* and the following point is valley*_i_*_+1_, until all the valleys have been connected. This represents the adaptative baseline (**Figure 3C**).

3. Adaptative baseline subtraction: After connecting all the valleys, we suggest to apply an adaptative baseline, in which *F*_min_ is substituted by the calculated adaptative baseline (see above, **Figure 3C**). Simply subtract the adaptative baseline from all the fluorescent values, and then divide it by itself, following the formula:

$$\text{SF fold change}=\frac{F_{i}-F_{\text{adaptBL}}}{F_{\text{adaptBL}}}$$

This is referred to as second-phase fold change, where *F_i_* is the fold-change fluorescence at a given time. *F*_adaptBL_ denotes the calculated lines correcting for changes in the baseline over the Ca^2+^ recordings.

4. Frequency: To calculate the frequency, we first identify each individual Ca^2+^ oscillation over a specific period of time. To detect islet Ca^2+^ waves, we suggest to impose a double threshold: (1) The islet fluorescent signal of individual peaks should present with at least an SF fold change >0.2 or 20% above the islet SF fold change (see above), which represent the activation point. (2) Only the activity sustained for at least 2 s should be considered as an islet Ca^2+^ peak or wave (**Figures 1B–D**). Once the signal returns and cross back, SF fold change <0.2, which represents the deactivation point or the end of the Ca^2+^ wave. Notably, if the fold-change fluorescent trace frequency is used to find the Ca^2+^ oscillations using these two parameters (20% deflection from the baseline and activity sustained >2 s), then only five Ca^2+^ waves are detected (**Figure 3E**). However, by correcting the changes in the baseline using the adaptative baseline, 15 Ca^2+^ waves are identified (**Figure 3F**). Finally, the frequency is calculated by dividing the number of Ca^2+^ waves (also referred to as Ca^2+^ peaks), over the specified period of time, i.e., 15 Ca^2+^ waves/1,200 s^−1^, or 0.75 Ca^2+^ waves per minute.
5. Second-phase peak amplitude: The average peak amplitude registered over all the Ca^2+^ oscillations in a specific period of time is referred to as the second-phase peak amplitude. After individual Ca^2+^ waves are identified, the maximum value of each Ca^2+^ peak (previously identified during the frequency analysis) is averaged and expressed as second-phase peak amplitude (**Figure 3F**).
6. Second-phase peak width: To calculate the second-phase peak width, calculate the average time that the islet spent as active per each peak, i.e., displaying fluorescent signal above 20% over the SF fold change. This coincides with the identification of the second-phase T_20-_*_i_*, here referred to as SP-T_20-_*_i_*, indicating the activation time for the *i*th Ca^2+^ wave. This should not be confused with FP-T_20_ or first-phase activation time. After identifying the islet T_20-_*_i_* for each Ca^2+^ wave, identify the time that the fluorescence returns to the baseline or is under <20% over the SF fold change or adaptative baseline. The total time spent as active is the individual peak width. Then, simply calculate the average for all the peak widths, and this is the second-phase peak width (**Figure 3F**).

### 5.6 Comments

The first peak after the initial increase of glucose at 11 mM usually initiates in WT (C57BL/6J background) after 4 ± 3 min, and lasts for 3–10 min (50). To differentiate activity vs. inactivity, we suggest a minimum threshold of 0.2 (fold change) or 20% increase above the baseline to be considered as active. The selection of this threshold value is discussed in more detail later (VI Functional subpopulation identification, D.2). This also coincides with the activation time or FPT_20_. However, for different sensors other than the GCaMP6 family, a higher or a lower threshold can be used to indicate activity. This method of quantifications can also be extended to intravital imaging, for example, to islets engrafted into the anterior chamber of the mouse eye. In those settings, obvious movements from the heart beating and the animal breathing generate subtle *Z*-plane movements. While these movements are usually restricted to <10 μm and consequently not moving away from the imaged cells, the fluorescent traces present “noise” from these movements. In those settings, the Ca^2+^ fluorescent trace smoothing helps to minimize the interference from the slight movement into the *Z*-plane *(*32, 48). Similarly, if the Ca^2+^ activity does not return to the recorded values at 3 mM after the first peak or first phase, we suggest to employ the adaptative baseline method, to compensate for the change in the baseline levels.

## 6 Functional subpopulation identification

The islet Ca^2+^ dynamics can be broadly grouped into two phases. During the initial response to the increase in glucose, or first phase, the islet presents a robust step increase in Ca^2+^ that can last for 3–10 min. After this initial peak, the islet transitions to what is commonly known as the second phase, in which spatially organized Ca^2+^ waves are displayed by the islet. This complex behavior is orchestrated by the functionally distinctive beta-cell subpopulation known as first-responders, hubs, and leaders. These subpopulations have been recently reviewed by Rutter et al. (4). Briefly, first-responders are involved during the first phase, while leaders and hub beta-cells are required during the second phase to initiate the Ca^2+^ waves and to connect the functional network of the islet.

### 6.1 Single-cell fluorescent trace extraction

1. To identify single cells, a time-series projection can be made to visualize individual cells. Briefly, in ImageJ, go to Image > Stacks > Z project, and select sum of slides. In the time-series projection, manually identify individual cells and use the polygon tool to add an ROI for each cell to the ROI manager (see above). During manual cell identification, first identify each single cell by observing the nuclei by a less intense GCaMP6f signal due to its cytoplasmic localization. Then, using the polygon tool, select around the nuclei, making sure to include 2–3 μm of cytoplasm, but making sure to not overlap the selection outside the cell. Save all the corresponding ROIs for the cells. From the ROI manager, click on “more” and click on “Multi Measure”. These are the fluorescence from all the frames for each cell. Save it as a csv file.
2. Go to “Analyze”, “Set measurements”, and select only “centroid”. In the ROI manager, click on “measure”. These are the coordinates of each cell. Save it as a csv file. These values represent the position of individual cells and are used for the topographic representation of the islet connectivity.
3. Similar to the islet, each single-cell fluorescent Ca^2+^ trace should undergo smoothing, baseline subtraction, and noise reduction.

### 6.2 Smoothing, baseline subtraction, and noise reduction

1. Similar to the islet, each single-cell Ca^2+^ fluorescent trace should undergo smoothing, baseline subtraction, moving baseline correction, and noise reduction (see 5 Islet global analysis, 5.2).

### 6.3 First-responders

1. The first-responders are found during the initial Ca^2+^ peak after glucose stimulation. These cells are characterized as 10% of the beta-cells with the fastest time of response during the first peak after glucose stimulation (31, 32). A critical difference between calculating the islet AUC and normalizing the fluorescence for finding the single-cell first-phase time of activation SC-FP-T_20_ is that the fluorescent trace is forced to fit in between 0 and 1. This is achieved by normalizing the fluorescent trace for each cell using a slightly different formula than the one used for the AUC:

$$\text{SC Normalized}=\frac{F_{i}-F_{\text{min}}}{F_{\text{min}}-F_{\text{max}}}$$

**Figure 4:**
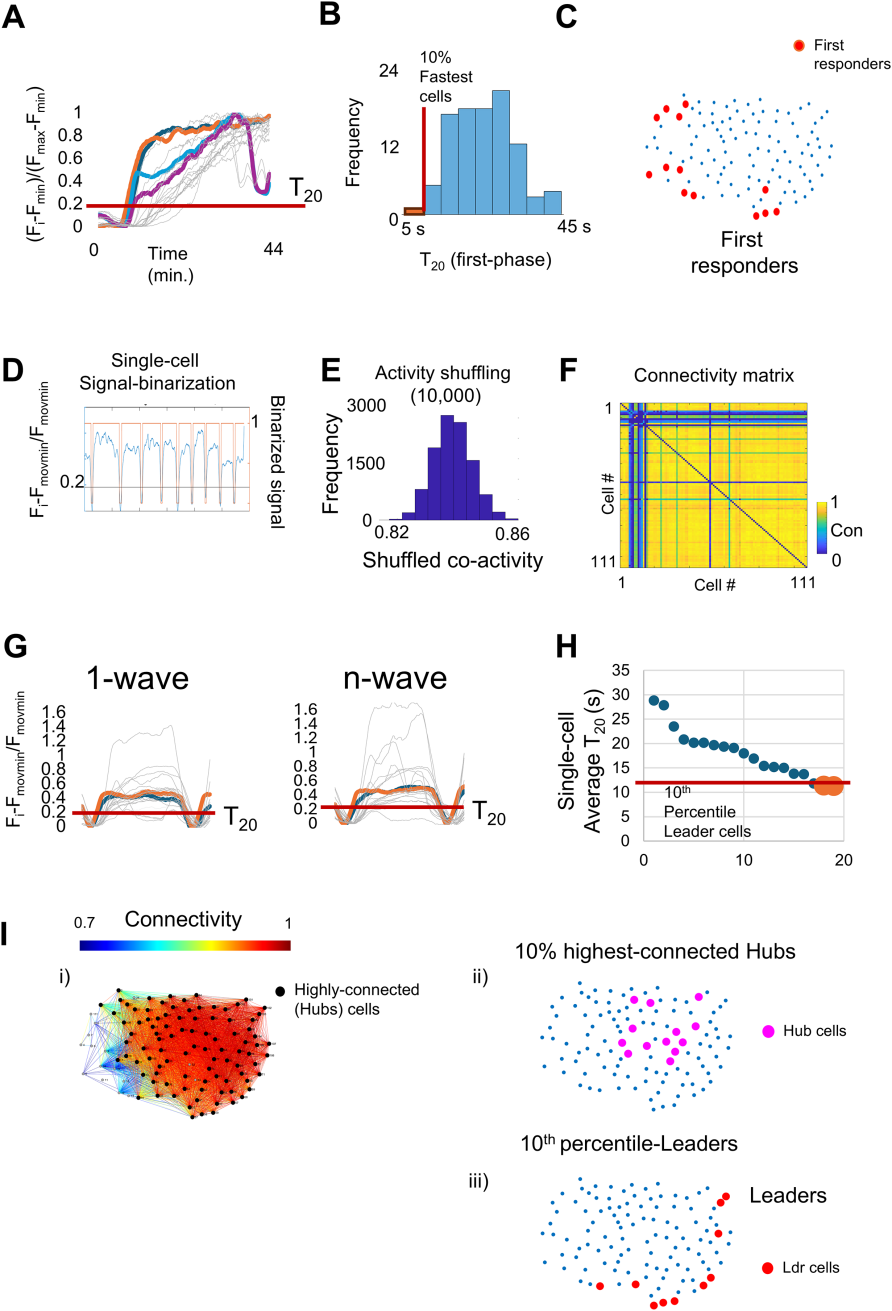
Functional subpopulation identification. **(A)** Single-cell fluorescent traces showing the T_20_ or activation time during the first peak. The colored traces represent the first three cells to show a response to 11 mM glucose. **(B)** Histogram showing the response time distribution for individual cells. The 10% cells that present the fastest T_20_ are identified as first-responders. **(C)** Plot showing the spatial localization for the identified first-responder beta-cells of the islet shown in **Figure 2A**; **Movie S1**. **(D)** The plot shows the fluorescent trace expressed as a fold change using the adaptative baseline (blue line). The red line depicts the fluorescent trace “binarization”, in which activity (SF Fold change >0.2) is represented as 1, otherwise as 0. **(E)** Histogram showing a cell-pair coactivity after shuffling 10,000 the recorded activity. **(F)** Coactivity matrix representing each cell pair coactivity in a color-key fashion. **(G)** Single-cell fluorescent traces of the first and last detected Ca^2+^ wave during the second phase, showing the identification of the T_20-_*_i_*. for each cell. **(H)** Average time of response (for all Ca^2+^ waves during the second phase) for individual cells. The fastest top 10th percentile are labeled as leader cells. **(I)** (i) Connectivity map showing the network topography and identified highly connected (hubs) cells (black dots). (ii) Top 10% best-connected hub beta-cell spatial identification (magenta dots) and (iii) leader spatial identification of the islet shown in **Figure 2A**; **Movie S1**, indicating the location of the leader cells (red dots).

This renders the single-cell fluorescent traces (SC Normalized), where *F_i_* is the fluorescence at a given time. *F*_min_ is the minimum and *F*_max_ is the maximum fluorescent intensity during the Ca^2+^-specific period to analyze. Of note, the *F*_min_ corresponds to the minimum value before the initial glucose response (**Figure 4A**).

2. Then, find the activation time for individual cells using the SC Normalized traces, or first-responders T_20_, or FR-T_20_, for each individual cell during the first peak (see 5 Islet global analysis, 5.4). FR-T_20_ is the time in which the individual cell shows an increase of 20% over the individual cell SC Normalized traces. Then, the 10% with the fastest time of activation are considered as the first-responders (**Figure 4B**; **Table 2**). Their spatial location can also be plotted (**Figure 4C**).

**Table 2:** Scripts for beta-cell functional subpopulation identification.

Subpopulation	Hyperlink	References
First-responders	https://github.com/jaelennox/FirstResponderCells_Kravetsetal https://github.com/jenniferkbriggs/Islet_Heterogeneity	(31, 34)
Hubs	https://zenodo.org/records/14042795/(Connectivity_GK-mCardinal.mlx) https://zenodo.org/records/14042795/(SciencesAdvancesPearsonCorrelationByphase.m) https://github.com/fionayongsuwern/10.1016-j.lfs.2023.121436 https://github.com/jenniferkbriggs/Functional_and_Structural_Networks.	(32, 34, 52)
Leaders	https://zenodo.org/records/14042795 (MVAR_Chabosseau.ipynb) https://github.com/fionayongsuwern/10.1016-j.lfs.2023.121436 https://github.com/jenniferkbriggs/Islet_Heterogeneity	(28, 33, 48)

The table provides several links for scripts developed to identified specific beta-cell subpopulation based on their Ca^2+^ dynamics.

### 6.4 Hubs and islet connectivity

1. During the second phase, the islet forms a functional network, in which individual cells display different levels of connectivity (3). Optogenetic hub silencing during the second phase or steady-state Ca^2+^ oscillation is enough to impair insulin secretion (27). We note that this class of “hub” cells was identified in studies using entrappable intracellular chemical probes (such as Fluo-4) in which connectedness, assessed as the extent of coactivity between cells, displayed a power law (“small worlds”) behavior in which a small number (5%–10% of total) were particularly highly connected (27). More recent studies using the GECI GCaMP6 fail to reveal compliance with a power law and hence the identification of a discrete “hub” population. The reasons for these differences are currently under investigation. Nevertheless, it is still possible using GCaMP6-expressing cells to identify a “highly connected” group that possess a high proportion (≥80%) of their connections with a strong coactivity coefficient (≥0.8) (52). The first step to identify these cells is to express the fluorescent traces as a normalized fold change (see 5 Islet global analysis, 5.2), using the following formula:

$$\text{SC Ffold change}=\frac{F_{i}-F_{\text{adaptBL}i}}{F_{\text{adaptBL}i}}$$

 This renders the single-cell fold change (SC fold change), where the *F_i_* is the fluorescence at a given time. *F*_adaptBL_*_i_* is the moving minimum or adaptative baseline for each individual cell. The valleys identified for the islet indicates the location to search from individual single-cell local minima. Then, calculate the individual single-cell adaptative baseline following the same procedure as for the islet adaptative baseline (see 5 Islet global analysis, 5.5).
2. Then, the SC fold change values are binarized: any time point 20% or above the adaptative baseline SC fold change is considered as “active” and represented by “1”. Any time point under the 20% threshold is considered inactive or “0”:

 f(x)={1 if x>0.2, otherwise 0}

where *f*(*x*) represents each individual SC fold change value and *x* is the binarization where values >0.2 are 1; otherwise, 0 (**Figure 4D**). This choice is based on an optimization approach: by varying the binarization threshold, we found that applying the 20% binarization threshold falls right into the elbow of the graph, to detect both average islet connectivity and % of highly connected (“hubs”) beta-cells (**Supplementary Figure 1**), thus representing the best balance between maximum noise suppression and minimum coactivity information loss. This optimization procedure can be utilized to determine the best binarization threshold when a different sensor is employed.

3. To calculate the coactivity coefficient for each pair of cells, a connectivity matrix is calculated, employing the following formula for each cell pair:

$$C_{ij}\frac{T_{ij}}{\sqrt{T_{i}*T_{j}}}$$

*T_ij_* denotes the total coactivity of each cell pair. *T_i_* and *T_j_* are the total activity time for the *i*th and *j*th pair of cells, respectively.

4. To discard connections that might arrive by chance, given the active time for each pair of cells, we calculate the connections that are expected by chance vs. the observed ones. This is achieved by shuffling (10,000 times) the observed activity for each pair and testing the significance of the observed connection versus the one expected by chance. The shuffling of the activity derives from the original cell pair activity. If a *t*-test, comparing the “shuffled” coactivity vs. the original one, displays a chance (>2 standard deviations, i.e., *p* < 0.01), cells are considered to be significantly linked (**Figure 4E**; **Table 2**, script 1).
5. To calculate the islet overall connectivity, simply remove the diagonal value of the connectivity matrix or the auto-connectivity values, then calculate the average for all *C_ij_* values or *C*_islet_ (**Figure 4Fi**). A low connected islet will present values of 0 < *C*_islet_ < 0.5; for medium connected islets, they will present values of 0.51 < *C*_islet_ < 0.75, and for highly coordinated islets, they will present values of 0.76 < *C*_islet_ < 1. A connectivity value of “0” means no coordination at all, while a connectivity value of “1” means perfect coordination. In practice, under normal conditions, healthy islets present a high degree of coordination, with an average connectivity coefficient between 0.76 and 0.95 (50).
6. Finally, to identify the hub cells, first filter the cells in which at least ≥80% of their connected pairs have a coactivity coefficient of ≥0.8. A topographic representation can be made using the Matlab wgPlot package (mathworks.com/matlabcentral/fileexchange/24035), using the connectivity matrix for the artists in a color-key fashion and the centroid to plot a dot indicating individual beta-cells. These thresholds are derived from studies using cell-entrapped probes such as fluo4 (27), wherein obedience to a power law was observed and 5%–10% of cells hosted the majority of the connections. However, we note that islets expressing the genetically encoded probes including GCaMP6, do not show an adherence to power law distribution (33). It is nevertheless convenient to describe the 10% best-connected cells as “hubs” (noting the above caveat) (**Figure 4Fii**).
7. To note: these calculations require substantial computing power: we recommend the use of a computer with at least 32 GB of RAM memory and a 2-GHz processor. Calculations can require up to 4 h for islets with >100 cells.
8. A macro for the above analysis can be downloaded from the sites given in **Table 2**.

### 6.5 Leaders

1. Similar to the highly connected cells, the leader cells are identified during the steady-state Ca^2+^ oscillations or “second phase”. To identify the leader cells, first calculate the single-cell second-phase T_20-_*_i_*, here referred to as SCSP-T_20-_*_i_*, or single-cell second-phase activation time (see 5 Islet global analysis, 5.4). The SCSP-T_20_ is the average time in which the individual cell shows an increase of 20% over the individual cell fold change moving baseline (**Figure 3G**).
2. From the peaks identified in the islet frequency islet, each Ca^2+^ peak is independently normalized using the same formula as before.

$$\text{SC fold change}=\frac{F_{i}-F_{\text{adaptBL}i}}{F_{\text{adaptBL}i}}$$

As previously stated, this renders the single-cell fold change (SC fold change), where *F_i_* is the fluorescence at a given time. *F*_adaptBL_*_i_* is the moving minimum or adaptative baseline for individual cells (see 5 Islet global analysis, 5.5). This helps to compensate for baseline changes over imaging and in between isolated Ca^2+^ waves (**Figures 2G**, **3**). By rescaling each Ca^2+^ wave individually, the time of response for each cell can be quantified. To identify the leader cells, calculate the average time of response over all the Ca^2+^ waves during the steady-state Ca^2+^ oscillations (**Figure 4G**). Then, those cells with an average time of response equal to or less than the 10th percentile are defined as leader cells. We have noted that leader cells are clustered in two or three different areas of the islet (**Figure 4H**, **Table 2**).

Macros from the above can be downloaded as detailed in **Table 2**.

### 6.6 Comments

For proper quantification of single-cell Ca^2+^ dynamics, it is recommended to exclude those cells that do not show Ca^2+^ increase after forced depolarization with KCl. This helps to reduce false positives, as extremely weak signals and unresponsive cells to KCl cannot be properly quantified. We also suggest to consider Ca^2+^ waves, when islet Ca^2+^ activity is sustained for ≥2 s. However, under specific conditions, Ca^2+^ wave frequency can be accelerated to sub-second transients (49, 50).

Of note, AUC and connectivity measurements quantify different aspects of islet Ca^2+^ dynamics. High connectivity does not always indicate a high AUC and *vice versa*. The AUC reflects the overall Ca^2+^ levels across imaging in respect to the 3 mM glucose baseline. However, connectivity is a coefficient of coordination (coactivity) across beta-cells. A connectivity coefficient of 1 indicates that a cell pair is perfectly coordinated and they are always active at the same time, a 0.5 connectivity coefficient indicates that a given cell pair is coordinated only 50% of the time they are active, and a 0 connectivity coefficient means they do not share any coordination.

We and others have employed Pearson correlation (*r*) to quantify similarity among the Ca^2+^ traces for individual beta-cells (3, 53, 54). Pearson correlation measures the strength of a linear relationship between two variables. A value of *r* > 0 indicates movement in similar directions, and a value of *r* < 0 indicates movement in opposite directions. Hence, it is common to only employ positive correlation values and perform the permutation test to discard those connections that might have arrived by chance (28, 32). We have applied the new methodology for signal binarization and coactivity measurement (see 6 Functional subpopulation identification). This led us to find that ~10% of the beta-cells present a high degree of connectedness in islets (27). Both of these methodologies refer to the significantly high degree of coordination as “connectivity”, which is the term used to describe the functional coactivity displayed by the beta-cells. We consider that binarization of the signal enables our connectivity measurements to be less sensitive to outliers. Of note, connectivity and Pearson correlation are not the only methods to evaluate functional connectivity; other methodologies to quantify connectivity metrics include but are not limited to Granger causality (28), mutual information, and entropy (55).

## 7 Representative results

We characterized the islet Ca^2+^ dynamics in response to glucose, including the global and individual beta-cell response to glucose. To this end, islets from the double transgenic mouse line Ins1Cre: GCaMP6^f/f^ were isolated (**Figure 1**) (28). The GCaMP6f Ca^2+^ sensor is expressed specifically in beta-cells, and it serves as a proxy for beta-cell function. After overnight recovery, 2 Hz confocal imaging was performed during a glucose ramp of 3, 11, and 25 mM glucose and a final membrane depolarization by 40 mM KCl (**Figures 2A–D**). The fluorescent intensity values were extracted using ImageJ (56). After quantification of the global parameters, we identify the first-responders, hubs, and leader beta-cells based on their specific Ca^2+^ dynamics (**Figures 2**, **3**; **Movie S1**).

For the first-responders, the first peak during the initial glucose increase can be plotted for each individual beta-cell. After fluorescence signal normalization, T_20_ can be calculated for each individual cell (**Figure 3A**). We defined the time of response T_20_ (s) as the time elapsed from the glucose increase (from 3 to 11 mM) for individual cells to present an increase of ≥20% in fluorescence intensity. The first peak is characterized by a fast step increase of Ca^2+^, which starts 3–10 min after glucose addition (**Figure 4A**). With a time resolution of 500 ms per frame, the individual time of response for each cell can be resolved, and a simple histogram showing the time of response can be plotted (**Figure 4B**). As noted by the fluorescent traces during the first peak, few cells present a Ca^2+^ response before the rest of the beta-cells. The 10% of cells with the fastest response time are the first-responders (**Figure 3C**; **Movie S1**).

After the initial peak, the islet transitions to steady-state Ca^2+^ oscillations. This is known as the second phase, and it is here where hub and leader beta-cells can be identified (**Figure 2B**; **Movie S1**). To identify hub beta-cells, the adaptative baseline fold change activity of each cell is binarized (**Figure 3D**). The coactivity of each pair of cells is calculated and tested against the shuffled coactivity (see 6 single-cell analysis, 6.2; **Figures 3E, F**). The average islet connectivity was 0.802. The hub cells are identified as those cells showing a high coactivity coefficient (≥0.8) in at least 80% of their connections. To identify the leader cells, the average time of response (T_20_) during the 25 mM glucose section was calculated (**Figure 4G**). Those cells with an average T_20_ equal to or less than the 10th percentile of the average response time from all the beta-cells are identified as leader beta-cells (**Figure 4H**). Finally, a topographic representation of the islet connectivity was generated using the coactivity matrix (**Figures 4I-i**) to also show the identified hubs. Similarly, the location of the identified 10% best-connected hubs cells are provided (**Figures 4H-ii**). Finally, leader cells’ location is provided (**Figures 4H-iii**). Notably, hub beta-cells are mainly found at the islet center, but first-responders and leader beta-cells are usually found at the islet periphery.

## 8 Discussion

The protocol described explores Ca^2+^ dynamics of beta-cells with single-cell resolution across imaging planes of the intact islet. After recording the Ca^2+^ responses to glucose, the first-responders, hubs, and leaders are identified. The optogenetic inhibition of the hubs interferes with islet Ca^2+^ coordination and insulin secretion (27). The laser ablation of first-responders and leader cells impairs the islet response to further glucose stimulation (28, 31).

The following are the important features of the protocol:

1. The general parameters of islet Ca^2+^ dynamics, including AUC, frequency, first- and second-phase amplitude, and width, are calculated. This protocol can also be used with Ca^2+^ dyes and AV-transduced islets.
2. We provide a specific methodology to quantify single-cell Ca^2+^ dynamics and how to identify each functional subpopulation. Hyperlinks to already published algorithms are also provided (**Table 2**).

We note that a major limitation of our protocol is the restriction to imaging a single confocal *Z*-plane to ensure high-speed data capture. This limitation could be overcome by using new faster imaging technologies, such as resonant scanner, multi-photon imaging, Z-Piezo, or light-sheet microscopy, to capture whole islet (or at least multiple cell layer) Ca^2+^ dynamics. Indeed, Jin et al. (2025) recently reported the usage of a light-sheet microscope to record the islet in 3D, which allowed them to achieve micron axial (*x*, *y*, *z*) resolution, which allowed them to achieve lateral resolution (x, y) resolution of ~200 µm^2^ and islet-deep axial (z) 132 µm resolution at 4 µm Z-steps (57). We consider that the protocol here described could be combined with 3D data to resolve whole islet Ca^2+^ dynamics at times shorter than 0.5 s per islet volume (48).

We note that hub beta-cells are defined as the most highly connected cells in a single plane within an arbitrarily selected plane of the islet, and it is under debate whether hub beta-cells can exert electrical control over the whole islet (58). However, single-cell-targeted optogenetic activation of beta-cells has provided evidence that some beta-cells have a disproportional capability to trigger Ca^2+^ increase in many cells beyond the immediate targeted beta-cell’s neighborhood (>10 μm away), in both fish and mouse islets (30, 32). It is still open how first-responders, hubs, and leader beta-cells exert their control over the rest of the islet beta-cells. These mechanisms might be orchestrated via gap junctions, ER-Ca^2+^ store mobilization, electrical coupling, and secretion of paracrine factors (33, 50). Indeed, leader cells are in close proximity to delta-cells, and it was recently reported that delta-cell optogenetic activation at a sub-maximal stimulatory glucose (7 mM) can trigger whole islet activity (33, 59).

Finally, most published experiments, even those conducted *in vivo*, have been performed in a relatively small window of time (<24 h). Therefore, the critical question whether these subpopulations represent transient states or functionally stable subpopulations remains to be answered (24). To address this question, we have conducted longitudinal studies *in vivo*, where we record the same set of cells over days and weeks, and we have found that leader and hub beta-cells are partially stable functional subpopulations (48).

Our strategy, which combines Ca^2+^ imaging and single-cell Ca^2+^ dynamics quantification, may prove useful to explore the functional networks of individual cells under control and experimentally diverse conditions.

## Data Availability

The datasets presented in this study can be found in online repositories. The names of the repositories and accession number(s) can be found in the **Table 2** (The scripts for specific subpopulation identification can be downloaded as indicated by in https://zenodo.org/records/14042795).
