# Correction: Pancreatic islet cell calcium ion imaging at single-cell resolution: functional identification of first responder, highly connected (“hub”), and leader beta-cells

**DOI:** 10.3389/fendo.2026.1872081

**Published:** 2026-05-13

**Authors:** Frontiers Production Office

**Affiliations:** Frontiers Media SA, Lausanne, Switzerland

**Keywords:** beta-cells, Ca^2+^, connectivity, first-responders, hubs, islets, leaders

The article type was erroneously given as **Review**. The correct article type is **Methods**.

The new **Data availability statement** section appears below.

“The datasets presented in this study can be found in online repositories. The names of the repositories and accession number(s) can be found in the Table 2 (The scripts for specific subpopulation identification can be downloaded as indicated by in https://zenodo.org/records/14042795).”

The new **Ethics statement** appears below.

“The animal study was approved by the CRCHUM Animal Facility (CIPA 2022–10,040 CM21022GRs). Colonies of Ins1Cre: GCaMP6f/f mice maintained on a C57BL/6J background, were fed a regular chow diet. The study was conducted in accordance with the local legislation and institutional requirements.”

The original version of this article has been updated.

