# Supplementary material for: Pancreatic islet cell calcium ion imaging at single-cell resolution: functional identification of first-responder, highly connected (“hub”), and leader beta-cells
Source: Front Endocrinol (Lausanne). 2026 Apr 15;17:1802510. doi: 10.3389/fendo.2026.1802510 (PMC13124488; doi:10.3389/fendo.2026.1802510)
Supplement: Supplementary file 1 [file Supplementaryfile1.docx]

**Supplementary materials**

**Supplementary Figure 1**

**
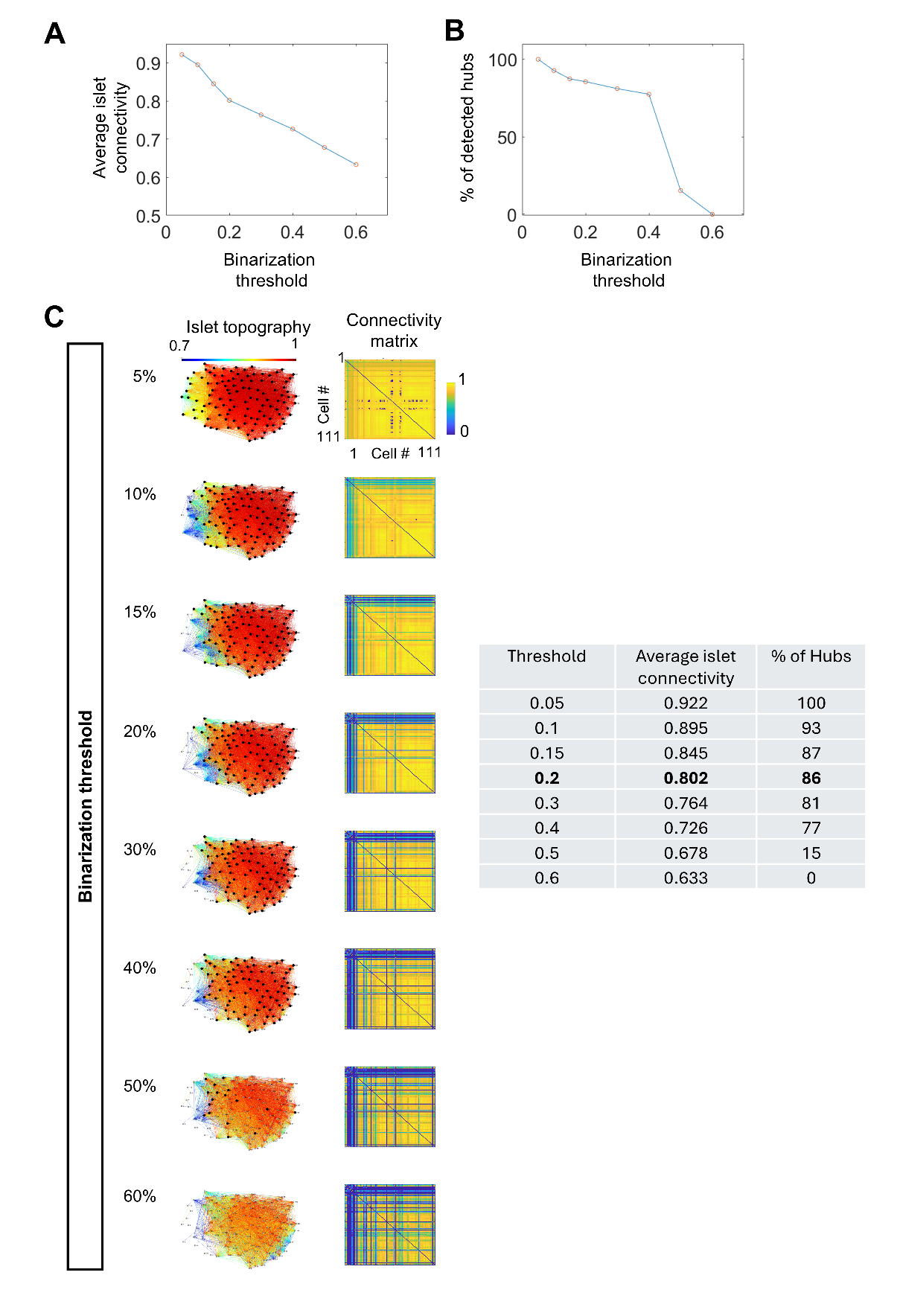
**

**Supplementary Fig. 1. Optimization of binarization threshold on average connectivity and “hub” cell detection**. **A)** Plot showing the effect of binarization threshold from 5% to 60% on average connectivity. **B)** Plot showing the effect of binarization threshold from 5% to 60% on hub cell detection. **C)** Islet functional topography and connectivity matrix given a specific binarization threshold. The table shows the exact numbers for each threshold. The table provides the specific number found for each binarization threshold. Reducing the activation threshold to 10% leaves noise to interfere to the posterior connectivity analysis. However, between 20% and 30% there is not a significant change of average connectivity and hub cell % identification. However, an exaggeratedly high i.e, 60% activation threshold causes that 0% of the cells to be identified as highly connected cells.

**Supplementary movies.**

**Movie S1. Islet confocal calcium imaging during a glucose ramp.** *In vitro* calcium imaging from transgenic C57BL/6J Ins1Cre:GCaMP6f^fl/fl^ isolated islet. The video was recorded at 2Hz with and a glucose ramp consisting in 3 mM, 11 mM and 25 mM glucose was performed. During the last 3 minutes, 40 mM KCl was added to force membrane depolarization.
